# Metropolitan Hospital for the Insane

**Published:** 1891-03-21

**Authors:** Francis H. Walmsley


### Metropolitan Hospital for the Insane.

By Francis H. Walmsley, M.D.

On the 25th ult., at a meeting of the Hospitals Association, an interesting discussion took place on the subject of "Hospitals for the Insane and Clinical Instruction in Asylums." It will be in the recollection of many that last year a committee of the London County Council reported that a lunatic hospital would be likely materially to increase the present knowledge of the nature and causes of insanity, and therefore ultimately to increase the means available for its prevention and cure. There can be little doubt that there prevails an uneasy feeling that the places in which the insane are received are too much asylums and too little hospitals. Is this so? In pursuing our inquiries let us exclude, as far as possible, whatever emotions the facts are calculated to excite, and attend solely to the interpretation of the facts. Our asylums as at present constituted are, and must inevitably be, "refuges" for stowing away all the wreckage of our social system, places where is thrown together everything in human nature troublesome and unsightly. The greater proportion of the inmates is made up of chronic and incurable cases. A large number of the most curable cases, those in fact that make up a considerable percentage of the recoveries, are patients suffering from the different forms of acute melancholia. These are they who have "fallen by the way," who have succumbed to the strain of a long period of anxiety and distress brought about by reverse of fortune, domestic affliction or other depressing influences, too often accompanied and aggravated by deprivation and want; they are reduced to a state of profound desolation of mind, sense of desolation so overwhelming that the unhappy sufferer welcomes death as the only escape from a life which to him has become intolerable. To brand such a one as "mad" in the ordinary acceptation of the term is unscientific, misleading, and brutal. The physical explanation of his condition is found in the disorder and defective nutrition of his "highest nerve centres"—a veritable starvation of the brain cells—and is a condition calling for treatment as active, watching as close as is given to a case of diphtheria or typhoid fever.

Turning to the question of clinical instruction in asylums, a recent writer says: "Owing to the want of special training the majority of medical men know no more about insanity than any lawyer or other intelligent layman." Here is a powerful argument in favour of utilising for clinical instruction the enormous mass of material at our command, and of throwing open our asylums to the large body of general practitioners and registered students. Surely it is not unreasonable to suggest that they should be accorded the opportunity, on one day at least in each month, of visiting the wards of the asylum with the view of familiarising themselves with the different forms of insanity, accompanied by the members of the resident staff, by whom they would be warmly welcomed, and every facility cheerfully afforded for clinical study and research. Further, it is most desirable that special and systematic training by a course of lectures and demonstrations should be given to asylum attendants. This is strongly advocated by the Medico-Psychological Association. By putting into practice such measures our lunatic asylums would approximate more and more in character to the hospital type.

I venture to say that if a poll of the medical officers, composing the permanent staffs of our lunatic asylums were taken, it would be found that, despite minor disagreements, the majority are united by the one common desire to see the establishment of lunatic hospitals on the lines of general hospitals. After all, the question is one which will be finally settled by an appeal to that "large sound round-about sense"—as Locke puts it—the heritage, not alone of any particular section of the community. Whether such hospitals for those suffering from acute mental disorders will be separate from the existing lunatic asylums and located in the metropolis and in the large towns in Great Britain, or whether they will be so constituted as to form a part of our present asylum system is a question that can be decided only by those who control the purse-strings.

